# Views and experiences of family physicians about Syrian refugee patients in Turkey: a qualitative research

**DOI:** 10.1017/S1463423621000190

**Published:** 2021-05-26

**Authors:** Duygu Ayhan Baser, Özge Mıhcı, Meltem Tugce Direk, Mustafa Cankurtaran

**Affiliations:** 1 Hacettepe University, Faculty of Medicine, Department of Family Medicine, Ankara, Turkey; 2 Hacettepe University, Faculty of Medicine, Department of Geriatrics, Ankara, Turkey

**Keywords:** experience, family physicians, refugee, Syrian, qualitative research

## Abstract

**Aim:**

The aim of this study was to describe the attitudes, views and solution proposals of family physicians (FPs) about primary healthcare problems of Syrian refugee patients. This study would be the very first study for Turkey that evaluates the attitudes, views and solution proposals of FPs about primary healthcare problems of Syrian refugee patients.

**Background:**

Following the anti-regime demonstrations that started in March 2011, the developments in Syria created one of the biggest humanitarian crises in the world and the largest number of asylum seekers continue to be hosted in Turkey. There are some studies evaluating asylum seekers’ access to healthcare services in Europe, and the common result is that refugees have free access to primary healthcare services in most countries; however, they face many obstacles when accessing primary healthcare services. While there are studies in the literature evaluating the situation of access to primary healthcare services from the perspective of asylum seekers; there are few studies evaluating the opinions/views of FPs.

**Methods:**

A qualitative methodology informed by the grounded theory was used to guide the research. A total of 20 FPs were interviewed face to face through semi-structured interviews, using 12 questions about their lived experience and views caring of refugee population. Interviews were analysed thematically.

**Finding:**

The following themes were revealed: Benefiting from Primary Health Care Services, Benefiting from Rights, Differences Between the Approach/Attitudes of Turkish Citizens and Refugees, Barriers to Healthcare Delivery, Training Needs of Physicians, Solution proposals. FPs reported that there is a need for support in primary care and a need for training them and refugees in this regard and they specified refugee healthcare centres are the best healthcare centres for refugees; however, the number of these and provided services should be increased.

## Introduction

Following the anti-regime demonstrations that started in March 2011, the developments in Syria created one of the biggest humanitarian crises in the world. On a population basis, Syria still has the highest number of refugees or asylum seekers population worldwide, with 12.6 million people (almost two-thirds of its population) (Cantekin, 2019). Turkey serves millions of refugees to the host due to its strategic location (Turkey shares the longest land border with Syria). By the end of 2017, there is a 21% increase in the population of refugees in Turkey and the largest number of asylum seekers continued to be hosting in Turkey. While this figure was 2.9 million in early 2017, it reached 3.691.333 as of 2019 (Republic of Turkey, 2019).

Since 2011, all Syrians who have been registered in Turkey with temporary identification numbers can benefit from the same level of primary and secondary healthcare services as Turkish citizens and the cost of this service will be billed to the governorship of the province. In order to prevent the problems faced by over three million Syrians while receiving health care and to reduce the workload of primary healthcare services, the Ministry of Health established ‘Refugee Healthcare Units’ in some regions where refugees live densely. In Refugee Health Centres, primary healthcare services are provided under the Public Health Directorates in Refugee Healthcare Units (Republic of Turkey, 2016). Arabic is the main language of Syria and Turkish is the main language spoken in Turkey. In Refugee Health Centres, healthcare providers who speak in Arabic are working, however, in family healthcare centres, not all healthcare providers can speak in Arabic.

Access to health care is crucial to the chances of life for asylum seekers who flee from conflict zones. In addition, access to health care is a basic human right regardless of immigration and compared to the general population, refugees who have more health risks (Fine, 2018). There are some studies evaluating asylum seekers’ access to healthcare services in Europe; these studies evaluated the views and experiences of asylum seekers and the common result is that asylum seekers use emergency health services more than the non-refugee population; this is due to the barriers to access to primary care. Refugees have free access to primary healthcare services; however, they face many obstacles when accessing primary healthcare services; lack of awareness of the nature and function of existing National Health System services, language barriers, interpreter problems, etc. They emphasised that there were insufficiencies regarding the issue (Crede *et al.*, 2018; Laban *et al.*, 2004; Cheng *et al.*, 2015; Priebe *et al.*, 2011; Kang *et al.*, 2019).

While there are studies in the literature evaluating the situation of access to primary healthcare services from the perspective of asylum seekers (Bhatia & Wallace, 2007; O’Donnell *et al.*, 2007; O’Donnell *et al.*, 2008; Osipovic, 2013); there are few studies evaluating the opinions/views of family physicians (FPs) who have a very important place in primary care in this regard (Begg & Gill, 2010; Lindenmeyer *et al.*, 2016); there is no study from Turkey. The ideas on primary healthcare needs of refugees of Turkish FPs (Turkey, which is the most refugee-hosting country in the world) should be very precious. It is very important to investigate the difficulties experienced by FPs about refugee patients, their experiences about them and their ideas about these services for the health policies to be developed in the new period. In this context, with this qualitative research, focusing on FPs, in-depth interviews with FPs about Syrian refugee patients service provision, their attitudes, the problems experienced and may be experience in service delivery and the solutions to health policies that can be developed as a result of the analysis of the interviews. So, the aim of the study was to evaluate the attitudes, views and solution proposals of FPs about primary healthcare problems of Syrian refugee patients.

This study would be the very first study for Turkey that evaluates the attitudes, views and solution proposals of FPs about primary healthcare problems of Syrian refugee patients.

## Methods

### Study design

The researchers conducted a qualitative study using semi-structured interviews with FPs to explore their attitudes views and challenges about primary healthcare problems of Syrian refugee patients. A qualitative methodology informed by the grounded theory was used to guide the research.

### Participants

The universe of the research consisted of FPs working in primary healthcare services in Ankara (which is the capital city of Turkey). Family medicine is a speciality in Turkey. In Turkey, all physicians who work in family healthcare centres are called as ‘family physicians’, those who are educated in family medicine departments for three years were called as ‘family medicine specialist’. Both FPs and family medicine specialists were included in the study. The sample was determined by ‘Convenience sampling’. There are 25 districts in total in Ankara province. FPs who work in primary healthcare settings from different districts of Ankara were included in the study. The study was conducted between March 2019 and February 2020. The interviewing process was continued until no new data were gained. A convenience sample of 20 FPs were selected by researchers.

### Data collection

Verbal and written informed consent and permission for audio recording were obtained from the participants before starting the interviews. The interview guideline was prepared. The interviews were performed between 1 March 2019 and 31 December 2019, and the analyses was ran from 1 January 2020 to 1 February 2020.

The questionnaire consists of two parts: the first part comprises questions to obtain demographic information, including age, gender, specialisation status, working year in the profession, the presence of registered refugee/asylum seekers in the population and the presence of refugee/asylum-seeking patients who are not registered, but who come to receive services. The second part of the questionnaire was semi-structured questions. The interview guideline was prepared, based on similar studies in the current literature with modifications and considering the situations faced by the researchers in their practice (Box 1). It was restructured after the pilot interviews. Three pilot interviews were conducted and some of the prepared questions were rewritten to be clearer.

The data were collected by two researchers, one observer and one moderator, in an environment where the participants could speak comfortably, in the family health centres and district health directorates, after the working hours of the physicians were over. Interviews were audio-recorded and transcribed verbatim. The interview time ranged from 25 to 40 min. During the in-depth interviews, the emotional state of the participants, body movements and facial expressions were recorded by the researchers. All of the important signs of body language were noted. The interviewing process was continued until no new data were gained. Saturation of data (the collection of data to the point where a sense of closure is attained because new data yields redundant information) was obtained after 20 interviews.

### Data analysis

All interviews were recorded digitally and the records were transcribed. The analysis included reading the transcript several times to gain an understanding of meaningful statements, and extraction of the themes present. Findings were then compared and discussed by the team until consensus on themes, theme clusters and categories was achieved. To test whether or not the data were correctly understood, the interview recordings were rechecked. Thematic codes were developed, and they were examined for their inclusive and exclusive aspects. Following analysis, the data were categorised into six themes.

### Ethical considerations

The ethical permission was obtained from the Non-Interventional Clinical Research Ethical Committee of Hacettepe University. Then, the participants were informed about the study and individual informed consent was obtained according to the principles of the Declaration of Helsinki.

## Results

A total of 20 FPs were interviewed for this study. Demographic data of the participants are presented in Table 1. The sample included 9 males and 11 females aged 27–60. As presented in Table 1, all of the participants (*n* = 20) were registered refugee patients and all of them could not speak Arabic, in which four of them could not speak English.

**Table 1.** Description of study participants

Participant No.	Age	Gender	Years after graduation	Specialist	The presence of registered refugee patients	The presence of refugee patients who want to register	English language skills	Arabic language skills
1	42	Male	19	Yes	Yes	Yes	Yes	No
2	47	Male	24	No	Yes	No	Yes	No
3	45	Female	20	No	Yes	No	Yes	No
4	30	Male	3	No	Yes	Yes	Yes	No
5	35	Female	11	Yes	Yes	Yes	Yes	No
6	32	Male	3	No	Yes	Yes	No	No
7	36	Female	11	No	Yes	No	Yes	No
8	38	Female	15	Yes	Yes	Yes	Yes	No
9	30	Female	6	No	Yes	Yes	Yes	No
10	58	Male	31	No	Yes	Yes	Yes	No
11	38	Male	13	Yes	Yes	Yes	Yes	No
12	56	Male	31	No	Yes	Yes	Yes	No
13	29	Female	3	Yes	Yes	Yes	Yes	No
14	36	Female	12	Yes	Yes	Yes	Yes	No
15	59	Male	33	No	Yes	Yes	No	No
16	36	Female	11	Yes	Yes	Yes	Yes	No
17	60	Male	35	No	Yes	No	No	No
18	44	Female	20	Yes	Yes	Yes	Yes	No
19	27	Female	3	No	Yes	Yes	No	No
20	35	Female	12	Yes	Yes	No	Yes	No

Six themes of the results were as follows: Benefiting from Primary Health Care Services, Benefiting from Rights, Differences Between the Approach/Attitudes of Turkish Citizens and Refugees, Barriers to Healthcare Delivery, Training Needs of Physicians, Solution proposals These themes are explained in detail.

### Benefiting from primary healthcare services

FPs had various opinions about the refugees’ current benefit from primary health care. The common idea of FPs was that if refugees request health service from any health centre, they can get service, if they do not, nobody can reach or want to reach them systematically.

Five physicians stated that Syrian refugee patients could not benefit from primary healthcare services sufficiently in our country and there is a problem with their registration and follow-up of registered refugees. And some FPs said that FPs are not proactively inviting them to their office. In their view:

“…If they (the refugees) demand, they definitely benefit, if they don’t, nobody is going to knock their doors.” (FP 5)

“…They (the refugees) cannot benefit from the health services what I have seen, they have no records, it is not clear whether they live in the country or not. If I vaccinate them where I will enter the vaccine code, I direct them to the district health center, they say they can not take care of it, and say let them go to the family physician, you cannot do it but how am I gonna do it?” (FP 6)

However, eight FPs emphasised that refugees benefit from health services and even more than Turkish citizens.

“…I think they face no obstacles anywhere, be sure everywhere is easier for refugee patients than Turkish citizens to enter, they can get their procedures done everywhere, they are welcomed and all their services are being provided.” (FP 1)

“…They benefit very well. They know the services, they receive the same service as Turkish citizens.” (FP 11)

Some physicians also stated that refugee healthcare centres meet the refugee patients’ primary care needs. According to FPs, these centres have been created to provide primary healthcare services to Syrians more effectively and efficiently, and to overcome the problems arising from the language and cultural barrier. There were also FPs who advocated for enhanced refugee healthcare centres and wanted to increase the number of these centres. There were FPs who stated that in addition to primary healthcare services, these centres should include internal medicine, paediatrics, gynaecology, oral dental health and psychosocial support services; imaging units and simple service laboratories (empowered refugee healthcare centres). Thus, it is aimed to increase access to services and to reduce the burden of the hospitals. Also, there were FPs who said that it would be appropriate to employ Syrian physicians in these centres if possible. In their view:

“…In this regard, I think that the number of outpatient clinics are sufficient in the refugee healthcare centres despite the high demand. I think that refugees who need secondary and tertiary health services, especially as a result of their application to public hospitals, cause intensity in the hospital and have problems in obtaining these services.” (FP 3)

“…Refugee healthcare centres have been very good. Their number should increase even more, but I think they need a systematized organisation.” (FP 13)

“…Refugee healthcare centres meet the primary need, but these people experience and create serious problems especially in other institutions. Therefore, Syrian branch physicians may be present in these centres if multidisciplinary is possible.” (FP 16)

### Benefiting from rights

Primary care services in Turkey are currently free to refugees. Universal Health Insurance for Foreigners residing legally for more than a year, asylum seekers and immigrants were included in the health insurance. All physicians stated that refugees should have the same health rights as the citizens.

“…The health right is a general right. They should also benefit. So they should be equal with the citizens of the country.” (FP 9)

Two of the FPs stated that it would be appropriate for refugees’ rights to be the same as the Turkish citizens, but these rights should not exceed the Turkish citizens:

“…Of course it must be the same as the Turkish citizen. They will live here now, but these rights should not exceed our citizens’ rights Otherwise there will be problems (laughs) and social exclusions would occur in every sense We are experiencing these.” (FP 13)

### Differences between the approach/attitudes of Turkish citizens and refugees

All FPs except two FPs stated that there is a difference between the approach of Turkish citizens and the approach and attitudes of refugee patients. Six doctors mentioned that the biggest difference in Syrian patients is the problems experienced in accessing the refugee patients for follow-ups (especially, vaccination, neonatal screening programme and pregnant follow-ups). FPs stated that the access information of registered Turkish patients is clear, but there is a problem with Syrians because of frequent change of residency, falsely given phone numbers, etc. In their view:

“…They change addresses very often. Especially address changes are it makes us tired. The vaccination, neonatal screening program and pregnant follow-up are very difficult. Therefore all of them should be registered” (FP 10)

“…There is a difference like this, we can easily access the contact information of the Turkish patients, there is no problem about the Turkish patients we want to reach with both the current population directorates and the current e-pulse information. But there is a problem in reaching a Syrian patient. The differences are in line with the demand, even if a Turkish patient does not demand, the family physician reaches him/her, but a Syrian patient needs to demand, he cannot access the service without demanding it.” (FP 15)

One physician mentioned the problem caused by the lack of past records of refugees as follows:

“…We often cannot reach medical history of foreign patients, so there is a difference in this regard. If we are going to be immunizing, of course there are differences because we do not know the previous vaccinations.” (FP 4)

Two FPs mentioned that the region where the refugees live was also effective and they stated that the reactions were different according to the region. While it could be easier to follow in the districts in small regions (rural), but a complete disaster in the big provinces.

### Primary healthcare needs of refugees and family physicians

All FPs stated that preventive health services were the major health needs of refugees, and these needs can be met in primary care by stating that:

“…As human, they also need whatever we need.” (FP 6)

“…They have all kinds of health needs. They are no different from the Turks, even have more demand.” (FP 12)

Three FPs emphasised that family planning needs are high due to their uncontrolled reproduction. Although the reason for this was not known exactly, there were physicians who stated that it was also thought-provoking that they refused the proposed family planning methods. In the FPs’ opinion:

“…I do not think that the health needs of Syrian refugees are very different from Turks, however; I think that providing services in family planning should be a priority and uncontrolled population growth should be prevented.” (FP3)

“…In my opinion, the most needed healthcare is family planning, they give birth non-stop. And also reproductive health.” (FP 17)

“…They are breeding uncontrollably, of course, as a physician, I think they most need family planning services.” (FP 19)

When all doctors participated in the need for immunisation. They talked about the potential infectiousness of refugees and the possibility of creating a risk for the community. Despite the past years, they thought that this was not fully regulated and especially because of their displacement and the inability to reach their addresses. Three physicians have been emphasized this topic more than others and, stated that:

“…If they are going to live in Turkish society, vaccination and immunization should be done to prevent disease in this society. If we don’t have a record, we have to do it all by assuming nothing is done, like from scratch; they are potentially infectious, we are being encountered with viral diseases that we cannot understand.” (FP 1)

“…I also think that their inclusion in vaccination programs should be a priority. I think that after this crisis in 2011, the strains that caused infectious diseases have also changed with the refugees who have settled in our country at a high number, and as a result, there are difficulties in the recognition of rash diseases.” (FP 3)

All FPs agreed that the majority of refugees had psychological problems because of their special conditions such as being in different countries, memories of war, loss of relatives. Many of the FPs would like multidisciplinary team in primary care including social workers and psychologists.

### Barriers to healthcare delivery

Language barriers were identified from FPs as one of the biggest problems in delivering quality care to refugees. Problems in finding interpreters, refugees not knowing Turkish and not trying to learn are amongst the problems shown. In the FPs’ opinion:

“…Yes, it’s an obstacle, we used to provide services with interpreters before the refugee healthcare centre is opened. Since I am not proficient in the language, I was having concerns about if the translation was correct and adequate when the healthcare service was being provided.” (FP 3)

“…Actually in the essence there is a language problem. I may not know how to explain things especially when using medical terms. Or I can not be sure that I understand her trouble.” (FP 9)

“…Interpreter and communication difficulties. There is no problem if there is an interpreter, but if not and we try to explain it with our own body language, wow. Firstly, I may not have understood his/her distress clearly, and secondly, I may not have fully expressed the way of treatment.” (FP 16)

Some physicians mentioned that the gender of the physician can be an important obstacle in the examination of women refugees. It was reported that male doctors suffer from this issue, especially in cases related to gynaecology or physical examination. Although they know that the fact that Syrians are mostly Muslims is the reason for this reason, they stated that they have great problems, especially in family health centres where there are no women physicians.

“…We do not have any female personnel, the woman patient does not get the examined, I ask why she came but she doesn’t understand. She looks meaningless and leaves like that.” (FP 6)

A physician stated that the differences in health systems between countries are an important obstacle with the following words:

“…The system in their home country is not like ours, their expectations may be different from what we give. We may have trouble in persuade our treatment.” (FP 7)

The two FPs mentioned that they do not know the legal process regarding the refugee patients and that this issue may cause problems. One of these two FPs stated that:

“…I do not know my rights in a legal issue or I don’t know their rights. This issue makes me very uneasy.” (FP 17)

### Training needs of physicians

Except for two physicians, all physicians stated that they should receive training about refugee patients on various subjects. Three physicians said that they may need to train language in terms of language proficiency. The vast majority of the physicians stated that they would like to receive training on vaccination/immunisation calendars, pregnancy follow-up programmes of refugees.

“…It is absolutely necessary, we do not know which vaccines are applied to the people coming from that region, something is being done in a sketchy manner, nobody is aware of each other and everyone is trying to do something on their own but this should be done by a single place, they should not go every healthcare centre because they are not being tracked, they go anywhere but you don’t know who is who and which is which.” (FP 1)

“…I think it’s necessary because; frankly, I do not know which screening programs should I start in a person who has entered the country in an existing refugee position.” (FP 15)

“…I think I need it. Frankly, there are diffucilties I have, I am explaining family planning, but I do not know what language they understands, do they know these things, do these methods exist in their country or in the same way, do they know about the vaccines I administer? For these reasons, education should be given to us and to refugees.” (FP 16)

### Solution proposals

The common opinion of all physicians on this issue is to increase the number of refugee healthcare centres. In addition to this, some physicians also have suggestions such as the outpatient clinic, the establishment of secondary/tertiary refugee centres or enhanced refugee healthcare centres. In their view:

“…The number of refugee health centres can be increased. District polyclinics can be established.” (FP 3)

“… Of course this is a business of the politicians but the important thing is an established system. In other words, I think these people should be managed not only in terms of primary care but also in terms of secondary/tertiary care centres which set up for them by physicians and healthcare professionals who know their language in different centres.” (FP 2)

“…Despite being hard to solve them all, healthcare services should be given to these people without being unfair to the Turks. I think polyclinics or family health centres specific to refugees should be established. The number of refugee health centres should be increased.” (FP 8)

“…The number of refugee health centres should be increased. Some can be selected among the family health centres. An icon can be added on it, indicating that it serves refugees. The thing here is not stigmatization. More educations can be given for example.” (FP 10)

## Discussion

The results of this study show that most of the refugee patients can benefit from primary health services without any problem; however, there is a serious problem in registration and follow-up of registered refugees. And FPs specified the need for support and training in this regard and according to them, refugees can be better followed up in refugee healthcare centres instead of family health centres.

According to some FPs in Turkey, refugees benefit more from healthcare centres than Turkish citizens; however according to some other FPs, Syrian refugee patients could not benefit from primary healthcare services in our country sufficiently, they mentioned the lack of records and follow-ups as the reason for this. Patient records are made to FPs in primary healthcare services in Turkey and monitoring is performed by FPs. This service also applies to registered refugees. The FPs reach the patients during their follow-ups for many follow-ups (such as baby, child, adolescent, pregnant, elderly, women aged 15–49) who are registered with the FP. However, follow-up cannot be performed unless there is an application by the patient for important follow-ups of a refugee who has not registered with a FP. This situation leads to a lack of records and follow-up of these people. For this reason, it is important for both individuals and public health to be registered by obtaining an ID number.

Registered Syrians in Turkey with temporary identification numbers can benefit from primary and secondary health services on the same level as Turkish citizens since April 2011 (Fine, 2018). However, non-registered Syrians in Turkey have limited access to primary and secondary healthcare services (Republic of Turkey, 2020). Refugee healthcare centres have been established in Turkey and these centres provide free screening tests, vaccinate children and babies within the scope of the Turkish immunisation programme, provide free iron and vitamin D supplements for pregnant and postpartum refugee women, distribute contraceptive materials and informative leaflets in Arabic to refugees (Ekmekci, 2017; Sahlool *et al.*, 2012). In this study, some FPs recommended that refugee healthcare centres were more appropriate for refugees to receive primary health care instead of family health centres and the numbers of refugee healthcare centres should be increased. In addition, some physicians stated that it would be more appropriate to expand these centres into centres that include the secondary health care by including the Syrian branch physicians. A total of 175 refugee healthcare units that were in 86 Refugee Health Centre and 17 provinces in Turkey were activated (Republic of Turkey, 2016). Increasing this number will increase the quality of service provided to refugees.

The UN Refugee Agency (UNHCR) points out that more than 63.3% of Syrian refugees are registered in Turkey (Fine, 2018). All physicians in this study also stated that refugees should have the same health rights as the citizens of the country they live in; however, they emphasise that these rights should not exceed the Turks, if there is an imbalance in rights, social discrimination may develop. Actually, health inequality can result in discrimination against refugees, and discrimination is a well-known social determinant of health, and have the potential to exacerbate negative health and well-being outcomes (Priebe *et al.*, 2011). There are some Turkish researches regarding the health needs of refugees (Cantekin, 2019; Sahlool *et al.*, 2012; Assi *et al.*, 2019). In many studies, refugees have not been identified as a homogeneous group and stated that they have different experiences and expectations of health and of health care and as a result, they have various healthcare needs. Women and children are often the most seriously affected people from migration. Screening and health promotion programmes tend to have a low uptake amongst refugee women and children. Family planning needs are high due to their uncontrolled reproduction. Women and men need to be presented with sexual health care and family planning. All these services can be done in primary care. Immunisation is another problem and healthcare need for refugees, especially for babies and children. Because of many reasons, very limited information was ultimately available on children’s vaccination status and needs. Today, Syrian infants and children are vaccinated within the scope of the Turkish immunisation programme in primary care. While limited rehabilitation services are provided in the health care for refugees, some international organisations provide rehabilitation via counselling services in Turkey (Sahlool *et al.*, 2012). According to this study, preventive healthcare services, outpatient services, immunisation, reproductive health services and rehabilitation services were stated as the major health needs of refugees. As mentioned above, the demands/needs of refugees in primary care are in a position to be met within the primary care system of our country. Most of the physicians included in the study stated that these needs were met in primary care services; there was no problem in the primary healthcare system, and the primary problem is due to the problems of refugees themselves.

Training of health workers in refugee issues has been identified as an important need in many studies, and has been requested by professionals who work with them (Trafford & Winkler, 2000; Burnett & Fassil, 2000). In this study, all physicians stated that they should receive training about refugee patients on various subjects, and the vast majority of physicians stated that they would like to receive training on vaccination/immunisation calendars, pregnancy follow-up programmes of refugees. And also physicians stated that the differences in health systems between countries are an important obstacle, and they want to learn about the health systems of refugees. Another important issue is the legal process regarding the refugee patients and FPs also request to be informed about this issue. However, in a study that aimed to detect some of the concerns of general practitioners (GPs) working in an urban environment about refugees, GPs had two different comments according to the number of refugees. GPs from low refugee and asylum seeker population areas stated that they had no specific training to deal with the healthcare needs of them, being ‘generalists’ they were able to adequately manage the medical needs of this population, without further training; GPs from high refugee and asylum populations concluded that although they might be able to deal with medical needs (Begg & Gill, 2010). In a study conducted by Katikireddi, GPs complained from their own knowledge and training about refugees (Burnett & Fassil, 2000; Katikireddi *et al.*, 2004).

In a systematic review conducted by Assi, they specified that the effectiveness of healthcare services for refugees was limited by language barriers, mobility of the refugees and some legal restrictions (Assi *et al.*, 2019). Parallel to Assi’s review, in this study, although serious problems have not been identified in the health services, the language barrier is a huge challenge in accessing health care for Syrian, lack of interdisciplinary rehabilitation services for refugees, the movement of Syrian refugees from one province to another also creates problems. Accessing the refugee patients for follow-ups is mostly very difficult, because of frequent changing of address, giving wrong phone number, etc. The vaccination, neonatal screening program and pregnant follow-up services are mostly affected services because it is difficult to control and monitor health. Problems in finding interpreters, refugees not knowing Turkish and not trying to learn are amongst the problems shown. Some physicians mentioned that the gender of the physician can be an important obstacle in the examination of women refugees. According to some studies, there is an increasing tendency towards the selection of same-gender physicians in the field of women health from conservative religious or cultural backgrounds (Roter *et al.*, 1999). Syrian women are mostly Muslims, which may have been effective in their choice. Although it is stated in the studies that women generally prefer female physicians in matters related to women’s health, in this study, it was observed that there is such a demand for FPs.

This study would be the very first study for Turkey that evaluates the attitudes, views and solution proposals of FPs about primary healthcare problems of Syrian refugee patients.

This research study was limited and specific to providing information about FPs’ experience caring for the refugees. The FPs identified a need for support in primary care and a need for training of them and refugees in this regard and they specified refugee healthcare centres are the best healthcare centres for refugees; however, the number of them should be increased and the services in it should be expanded.
